# Leptin, CD4^+^ T_reg_ and the prospects for vaccination against *H. pylori* infection

**DOI:** 10.3389/fimmu.2012.00316

**Published:** 2012-10-15

**Authors:** Anna K. Walduck, Dorit Becher

**Affiliations:** Department of Microbiology and Immunology, University of MelbourneParkville, VIC, Australia

**Keywords:** *Helicobacter pylori*, vaccine, regulatory T cell, leptin

## Abstract

*Helicobacter pylori* infection induces chronic inflammation which is characterized not only by infiltrations of inflammatory cells such as neutrophils and CD4^+^ T cells, but also significant populations of regulatory T cells (T_reg_). These cells are important for disease pathogenesis because they are believed to contribute to the persistence of the infection. Despite encouraging results in animal models, the prospects for an effective *H. pylori* vaccine are currently poor because of generally disappointing results in preclinical and phase 1 trials. As a result, a current major focus of basic research on vaccination is to better understand the mechanisms regulating the inflammatory response with the view it can inform future vaccine design. Our studies in this area have focused on gastric CD4^+^ T_reg_ in vaccinated mice, and raised the hypothesis that adipokines in particular leptin are involved the establishment of a protective gastric immune response. Here we discuss the hypothesis that vaccination deregulates T_reg_ responses in the gastric mucosa, and that this process is mediated by leptin. We propose that reduced suppression permits a protective sub population of *H. pylori*-specific CD4^+^ T cells to exert protective effects, presumably via the gastric epithelium. Evidence from the literature and experimental approaches will be discussed.

## INTRODUCTION

### *H. pylori* INFECTION INDUCES BOTH A STRONG INFLAMMATORY CD4^+^ T CELL AND CD4^+^ T_reg_ RESPONSE

*Helicobacter pylori* chronically infects the mucus gel layer of the human stomach. This pathogen is highly adapted to the gastric niche and the natural immune response does not clear infection (Blaser, 2004). Most cases of *H. pylori* gastritis are sub-clinical, and the inflammation leads to symptomatic gastritis in only about 10% of infected persons, the implications of asymptomatic infections remain unknown (Robertson et al., 2003). *H. pylori* pathogenicity is dependent on both host and bacterial factors and the mechanisms are complex and remain to be explained clearly (Blaser, 2004).

*Helicobacter pylori* gastritis is characterized by strong infiltrates of CD4^+^ T cells, Neutrophils and B cells (D’Elios et al., 1997; Strömberg et al., 2003) in the gastric mucosa. When one considers the high numbers of persons chronically infected with *H. pylori* worldwide, and that a relatively low proportion of these develop symptoms, it might be seen as circumstantial evidence that this inflammation is under tight regulatory control. Studies in biopsies from infected patients and experimental animals have supported the importance of suppressor or regulatory T cells (T_reg_) (CD4^+^/CD25^+^/FoxP3^+^) in *H. pylori* pathogenesis. Indeed relatively high numbers of *H. pylori*-specific T_reg_ are present in the stomach of asymptomatic *H. pylori*-infected patients (Lundgren et al., 2003; Goll et al., 2007) and that these suppressed memory T cell function *in vitro*. Similarly, studies in experimentally infected mice showed that T_reg_, suppressed proliferation of CD4^+^ T cells, and that this probably contributes to the persistence of the pathogen (Raghavan and Holmgren, 2005; Rad et al., 2006). In addition, these CD4^+^ T_reg_ have been shown to be specific for *H. pylori* antigens in humans (D’Elios et al., 1997) and mice (Raghavan et al., 2004). Further, Enarsson et al. (2006) reported antigen-specific functional T_reg_ in the mucosa of patients with gastric tumors, it is noteworthy that greater numbers of T_reg_ were detected in tumors as opposed to non-tumor tissue. In this circumstance, T_reg_ may suppress natural protective anti-tumor mechanisms.

It is likely therefore that in most infected persons, *H. pylori* gastritis is tightly controlled by a complex balance of pro-inflammatory and suppressive T cells, and only when this balance is shifted by other factors do overtly pathogenic mechanisms move into action. The impact of these mechanisms is evident in the prevalence and clinical significance of gastric and duodenal ulcer disease, and gastric cancer. Understanding the factors influencing T_reg_ function in the stomach will be one of the keys to progress in therapy and vaccination against *H. pylori*.

## GASTRIC T_reg_ AS AN OBSTACLE TO VACCINATION

The reports of successful immunization in animal models lead to early enthusiasm for a vaccine, and a variety of strategies, both oral and parenteral have been shown to be effective at reducing, but in general not completely clearing colonization (e.g., Aebischer et al., 2008b; Müller and Solnick, 2011). While there are some reports of sterilizing protection in mice (Kleanthous et al., 2001; Garhart et al., 2002; Panthel et al., 2003b) and in the gerbil model (Jeremy et al., 2006), the overwhelming majority of reports report 1–3 logs reduction in colonization, but not clearance (reviewed in Zhang et al., 2011). Data from animal studies have showed that vaccine-induced protection is accompanied by increases in gastritis in vaccinated mice (Ferrero et al., 1994; Sutton et al., 2001; Garhart et al., 2002), prompting the general belief that strong inflammation is responsible for protection.

Early vaccine trials in human volunteers were disappointing with regard to efficacy, but showed that oral vaccination with recombinant antigens was at least feasible (Kreiss et al., 1996; Kotloff et al., 2001). Limited immunogenicity and a relatively high incidence of adverse effects were noted. Recent vaccine trials have reported good immunogenicity, e.g., a mixed antigen vaccine administered parenterally was immunogenic in *H. pylori* negative volunteers (Malfertheiner et al., 2008). However to date there have been only three trials in infected persons: two trials in *H. pylori*-positive volunteers (Kreiss et al., 1996; Kotloff et al., 2001) and one in prophylactically immunized and experimentally challenged volunteers (Aebischer et al., 2008a). Although the vaccines were reported as safe, significant, if minor adverse effects were noted in all studies, and only very limited evidence of protective effects was obtained.

The impetus for vaccine development was lost because of the generally discouraging results discussed above. Progress in our understanding of the regulation of gastric inflammation, and pre clinical testing of new antigen combinations and adjuvant formulations are now a major focus. Questions have also been raised as to whether vaccination is even feasible, and indeed the obstacles are seemingly great. Given that the local gastric inflammatory response in natural infection is apparently so tightly regulated, a successful therapeutic vaccination strategy would need to overcome this regulation, without tipping the balance toward activating severe gastritis. T_reg_ may not be a hindrance to prophylactic vaccination, as they might not be expected to appear until several weeks/months after infection. Both prophylactic and therapeutic vaccination must also direct a protective response via the epithelium to clear an essentially extra cellular, motile, luminal pathogen.

Interest in an *H. pylori* vaccine has been rekindled with reports of increasing antibiotic resistance (particularly to metronidazole and clarithromycin; Graham et al., 2007). Vaccination experiments in knock-out mouse strains showed that in the absence of functional CD4^+^ T cells, vaccination was not protective (Ermak et al., 1998; Pappo et al., 1999). In addition, adoptive transfer of T cells (CD4^+^) from vaccinated mice conferred protection in *H. pylori*-challenged recipients (Mohammadi et al., 1997; Lucas et al., 2001). Although this model is not entirely straightforward and we have been able to reproduce these results in C57BL/6 mice in independent experiments in one laboratory but not after moving to another (Walduck and Becher, unpublished observations). The data from CD4-deficient mice are most compelling and clearly these cells are instrumental in mediating protection. On the other hand, T cell driven immune responses are also implied in induction of gastric neoplasia (Enarsson et al., 2006; Sayi et al., 2009). Indeed Mueller et al. (2009) have shown evidence that the protective and pathogenic effects of T cells may be inseparable.

Others have suggested that there may be phenotypically different sub-populations of CD4^+^ cells that have protective effects (Blanchard et al., 2004; Walduck et al., 2004). Evidence to support this will be discussed here.

With the importance of T_reg_ in *H. pylori* gastritis in mind, and the potential obstacles for vaccination, there is regrettably almost no information from the published human vaccine trials to date on the impact of vaccination on T_reg_. That T_reg_ may not interfere with prophylactic vaccination was supported by the report of Aebischer et al. (2008a), where FoxP3 positive cells were only detected in the mucosa in *H. pylori*-challenged volunteers after 3 months. There was however no significant effect of vaccination, at least up to 3 months when the study ended (Aebischer et al., 2008a, supplementary information).

Studies in animal models show significant populations of T_reg_ in *H. pylori*-infected mice (Raghavan et al., 2004), including in the stomach (Becher et al., 2010). Other studies showed that depletion of T_reg_ led to worsened gastritis (Kaparakis et al., 2006). Depleting T_reg_ would be expected to improve vaccine efficacy by permitting increased pro-inflammatory responses, and this was reported by Zhang et al. (2008) who employed antigen pulsed DCs for vaccination. There are no other published reports of this phenomenon. There remain no published data from vaccination studies employing the mouse strain developed by Rudensky and colleagues (Kim et al., 2007) where depletion of T_reg_ can be selectively induced by injection of diphtheria toxin. This model offers the best possibility to determine the role for T_reg_ *in vivo*.

It should, however, be noted that alongside the reports of exacerbated gastritis in vaccinated mice, there are also numerous reports of effective vaccination (1–3 logs reductions in colonization) in other strains of mice, e.g., BALB/c, where gastritis minimal, or undetected by traditional methods (Gómez-Duarte et al., 1998; Lucas et al., 2001; Panthel et al., 2003a). This implies that gastritis *per se* is not they key requirement for a protective response and that is the first suggestion that small protective sub-populations are present.

We have investigated CD4^+^ and CD4^+^ T_reg_ function in vaccinated C57BL/6 mice and compared gastric infiltrates to those in the circulation, spleen and lymph nodes. We found not only increased numbers of CD4 in the stomachs of vaccinated mice (as has been reported by others) but also decreased proportions of the T_reg_ sub-population of CD4^+^ cells in the stomach. These data imply that vaccination reduced suppression of CD4^+^ T cell numbers and function, and predicts that these cells will have a higher proliferative capacity. This was indeed observed in *in vitro* studies (Becher et al., 2010).

The lack of requirement of antibody (Ermak et al., 1998) and many inflammatory cytokines including IFNγ, for vaccine mediated reductions in colonization, was unexpected in light of the strong Th1 response induced both infection and vaccination (reviewed in Aebischer et al., 2008b). Gene expression studies were an alternative attempt to identify new pathways and mechanisms involved in protection (Mueller et al., 2003a,b; Rahn et al., 2004; Walduck et al., 2004).

One of the novel signature genes identified in these studies was adipokines – a family of genes associated with fat cells (Mueller et al., 2003a; Walduck et al., 2004). *Adipokines* are defined as cytokines secreted by adipose tissue, many of which have endocrine activity. This group includes leptin, chemerin, resistin which have pro-inflammatory activity, and adiponectin which has anti-inflammatory activity (La Cava and Matarese, 2004; Goralski et al., 2007; Wilk et al., 2011).

## A ROLE FOR LEPTIN IN REGULATING INFLAMMATION

Leptin (Ob – the obese gene) is an adipocytokine that links nutrition with immunity. Adipokines are produced by mature adipocytes and other cell types (e.g., T cells, T_reg_, epithelial cells; Siegmund et al., 2002). Leptin (also known as Ob, the obese gene) is the best-studied adipokine to date, and acts as a hormone that regulates the perception of hunger, and therefore food and energy intake (Fantuzzi, 2005). Leptin has since also been implied in cytoprotection (Konturek et al., 2001), bone mass control (Elefteriou et al., 2004), and immune functions (Lord et al., 1998; Fantuzzi et al., 2005), including the effect of nutritional status on immune dysfunction (Papathanassoglou et al., 2006).

**FIGURE 1:**
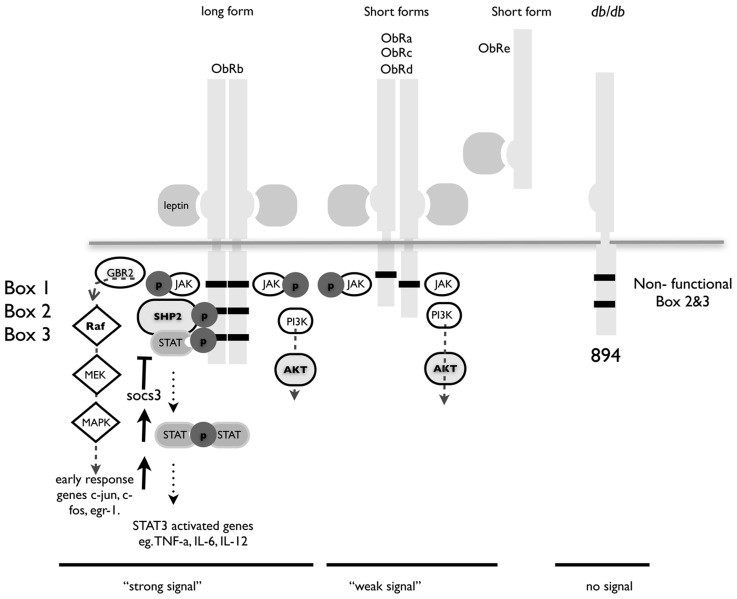
**Structure of leptin receptor isoforms.** Ob-Rb contains the longest intracellular domain, which is crucial for leptin signaling. Ob-Ra, Ob-Rc, and Ob-Rd contain only short cytoplasmic domains. Ob-Re is truncated at position 830. Box 1 is a JAK docking site, boxes 2 and 3 are STAT docking sites. A point mutation at position 894 in the long form of ObR in the *db/db* mouse renders it incapable of signaling. The *db/db* mouse is a well-characterized model for type I diabetes and these congenic mice have a single nucleotide mutation that result in splicing of a non-functional version of the major signaling isoform Ob-Rb.

The leptin receptor is a single transmembrane protein that is similar in structure to the gp130 family of receptors. Binding of the 16-kDa leptin protein triggers the receptor to form homodimers and induces signaling principally via the JAK/STAT pathway (La Cava and Matarese, 2004; **Figure 1**).

Ob-Re binds leptin but has no intracellular domain and although there is a report that it can initiate signaling, is thought to act chiefly as a method of storing leptin and as such prevents signaling and transport of leptin (Tu et al., 2010). Leptin receptor-signaling deficient mice are obese, and diabetic and display a marked increase in the number and suppressive function of T_reg_ cells (Taleb et al., 2007). Leptin can also directly regulate T_reg_ function by acting as a strong has a strong inhibitor of proliferation (De Rosa et al., 2007).

Leptin also acts as a chemo-attractant for neutrophils and macrophages (Montecucco et al., 2006; Gruen et al., 2007), these cells are also present in high proportions in vaccinated stomachs (Becher et al., 2010). Both macrophages and neutrophils are thought to migrate in response to local leptin gradients in tissues. Interestingly, *in vitro* stimulation via the leptin receptor induced migration, but not activation of neutrophils and rendered them unresponsive to “classical chemotaxins” such as IL-8 (Montecucco et al., 2006). Conversely, leptin stimulation induced migration and activation (caused Ca^2^^+^ influx), but did not interfere with the ability of a macrophage cell line to respond to MCP-1 (Gruen et al., 2007).

## LEPTIN RECEPTOR SIGNALING IS REQUIRED FOR VACCINE-INDUCED PROTECTION

As part of the follow-up from gene expression studies, we tested prophylactic vaccination in leptin receptor signaling deficient C57BLKS Cg Lepr*db/db* mice, and found that they were not protected by prophylactic vaccination (Wehrens et al., 2008). This was in spite of developing (mild) gastritis and *H. pylori*-specific antibody. While the latter does not necessarily suggest a protective response, it does indicate that the *db/db* mouse is immunocompetent. We hypothesized that leptin signaling was required, not for mounting an *H. pylori*-specific response, but rather for transmitting the protective “ message” from the mucosa to the gastric epithelium.

Taken together with the evidence from the literature on the influence of leptin signaling on T_reg_, macrophages, and neutrophils, it seems feasible that leptin signaling may also provide a link between CD4^+^ function and protection in wild-type mice.

Based on the literature, and results of our own experimental data discussed below, we proposed that leptin plays a key role in signaling between protective T cells and gastric epithelium.

Hypothesis:

- That strong gastritis is not required for a protective immune response in vaccination – a small sub-population with a “protective” phenotype is sufficient.
- That leptin signaling regulates CD4 cell function by interfering with the function of T_reg_ and inflammatory cells in the stomach.

The above hypotheses lead to the following predictions:

Successful vaccination will be observed in mouse strains/models where gastritis is mild/undetectable.

BALB/c mice develop weak, or delayed gastritis after *H. pylori* infection (Lee et al., 1997; Panthel et al., 2003b). After vaccination, BALB/c mice develop increased (although mild) gastritis and are protected to similar level as strains that develop strong gastritis such as C57BL/6 (Gómez-Duarte et al., 1998; Koesling et al., 2001; Panthel et al., 2003b; Walduck et al., 2004; Sutton et al., 2007). The reduced gastritis observed in BALB/c mice might be expected to be due to the Th2 bias and increased proportion of T_reg_ found in this strain (Sacks and Anderson, 2004), however, Kaparakis et al. (2006) reported that depletion of T_reg_ using anti-CD25 antibody did not affect the intensity of gastritis, but the cytokine secretion pattern. The use of anti-CD25 antibodies could also have depleted other activated T cell populations and so the approach using specific *in vivo* depletion of T_reg_ discussed above will be required to clarify this question.

It is interesting to note that while CBA mice, which also develop only mild gastritis, were protected by prophylactic vaccination, therapeutic vaccination was ineffective (Sutton and Wilson, 2004). It would appear that additional genetic factors have a role to play in gastric inflammation.

If a small sub-population is responsible for protection, then a distinct phenotype of the protective will be recognizable, and small numbers will be required for protection.

The microarray studies that display a specific gene expression signature in protected mouse stomachs are indirectly suggestive of this protective phenotype (Mueller et al., 2003b; Rahn et al., 2004; Walduck et al., 2004). Further, analysis of gene expression patterns in biopsies from vaccinated human volunteers also suggested a specific expression pattern and “T cell footprint” (Aebischer et al., 2008a).

Analysis of gene expression patterns in lymphocyte sub-populations isolated from gastric tissue of vaccinated and control mice will provide more specific information on the “protective program” induced in these cells.

Adoptive transfer studies where splenocytes or CD4^+^ cells from vaccinated mice were transferred to recipients provided confirmation for the importance of these cells for protection, relatively small numbers of cells are required to achieve this effect (in the order of 5 × 10^6^). These adoptively transferred cells do migrate to the gastric mucosa and are detectable in small numbers (Aebischer et al., 2008b; Becher and Walduck, unpublished observations).

That vaccination will be ineffective in the absence of leptin

As discussed above, we reported the requirement for functional LepR signaling for protection in 2008 (Wehrens et al., 2008). The result was observed using two different prophylactic vaccination strategies. We have since also shown this in *db/db* mice backcrossed onto a C57BL/6 background (Becher et al., in preparation).

*If our previous observations hold, then leptin will impact CD4*^+^ T cells and T_reg_ proliferation and function.

The first suggestion for this comes from the analysis of gene expression patterns in the stomachs of vaccinated and non-protected *db/db* mice. For the most part, we observed the same genes were expressed in protected wild-type as obese mice, but interestingly, genes were more strongly up- or downregulated in obese *db/db* mice (Wehrens et al., 2008). This is suggestive of a lack of appropriate regulation or control of the inflammatory response in the absence of leptin signaling. Our observation is also in keeping with the reported role for LepR signaling in T_reg_ function. De Rosa et al. (2007) proposed that T_reg_ control their own function in an autocrine fashion, by expressing both leptin and leptin receptor.

**FIGURE 2:**
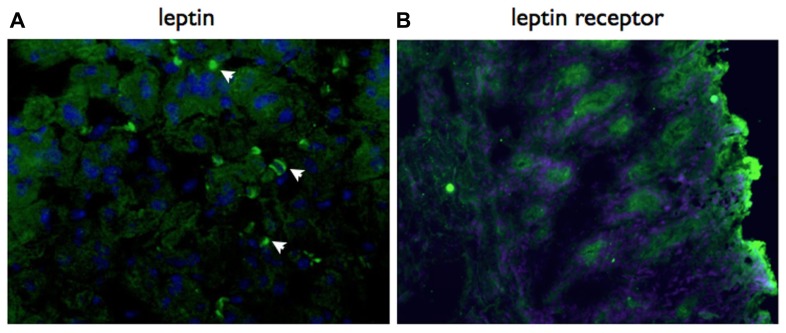
**Both leptin and leptin receptor are expressed locally in the gastric mucosa.** Immunohistochemistry using specific antibodies, detected with a Cy2 labeled secondary antibody. Both leptin **(A)** and lepR **(B)** are expressed in the gastric mucosa of vaccinated mice at day 21 post-challenge with *H. pylori* SS1. Mice were vaccinated with recombinant *S. typhimurium* expressing *H. pylori* urease as previously described (Becher et al., 2010). Original magnification ×20.

Based on the results of our previous descriptive studies on local gastric responses (Becher et al., 2010), we predicted that the role for leptin would be in a local gastric signaling network. Consistent with this proposal, we have detected both gastric epithelial cells and infiltrating lymphocytes express leptin receptor in *H. pylori*-infected mice (**Figure 2**).

As a result of studies in models of both autoimmune and infection, leptin is now accepted to play an important role in regulating the Th1/Th2/T_reg_ balance (Procaccini et al., 2012), and in this manner to regulate disease. A number of recent publications point to the importance of leptin in T_reg_-associated disease which may well be applicable to the *H. pylori* system. T_reg_ play an important role in the complex inflammatory response in atherosclerosis (Gotsman et al., 2007). Huertas et al. (2012) reported that dysfunctional endothelial cells from idiopathic pulmonary arterial hypertension (IPAH) patients produced increased levels of leptin, and a higher proportion of their T_reg_ expressed leptin receptor. The authors hypothesize that leptin disregulates T_reg_ and contributes to disease (Huertas et al., 2012). In addition, studies in mouse models of atherosclerosis revealed a critical role for leptin in the alteration of the regulatory immune response. Defective LepR signaling improved T_reg_, function and led to dramatic decreased in lesion size (Taleb et al., 2007).

Given that leptin is ubiquitous in normal serum, *in vitro* studies on CD4^+^ T cell and T_reg_ function will not have addressed the situation of limiting leptin concentration, this point has also been raised by other authors (De Rosa et al., 2007). Available leptin concentrations in the tissue may well be lower than in standard tissue culture media. We are currently addressing this issue by examining the proliferative and suppressive ability of *H. pylori*-specific T_reg_ under leptin-limiting conditions.

**FIGURE 3:**
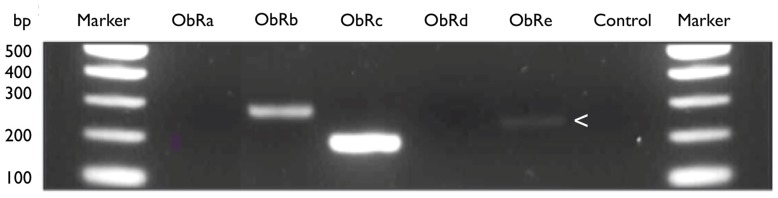
**LeptinR isoforms Ob-Rb, Rc, and Re by murine CD4^+^ T cells.** Expression of leptinR isoforms by RT-PCR. CD4^+^ T cells were isolated and purified by negative selection from the MLN of *H. pylori *unvaccinated, infected female C57BL/6 mice. Equal amounts of MLN cDNA were analyzed for expression of Ob-Rb (295 bp), Ob-Rc (205 bp), and Ob-Re (257 bp, expression of Ob-Re is indicated with an arrow head). Positive control HPRT (399 bp), no RT control (control).

Because commercially available antibodies detect all receptor isoforms, there is also little information on the expression of the various isoforms of on immune cell populations. We speculate that leptins’ influence on T_reg_, CD4 and neutrophil function is orchestrated by expression of different receptor isoforms. Our preliminary analyses using an RT-PCR approach on sorted cell populations have shown that at least three different isoforms of LepR are expressed by CD4^+^ cells alone (**Figure 3**). We anticipate that further studies will reveal this repertoire may also be tissue-specific.

**FIGURE 4:**
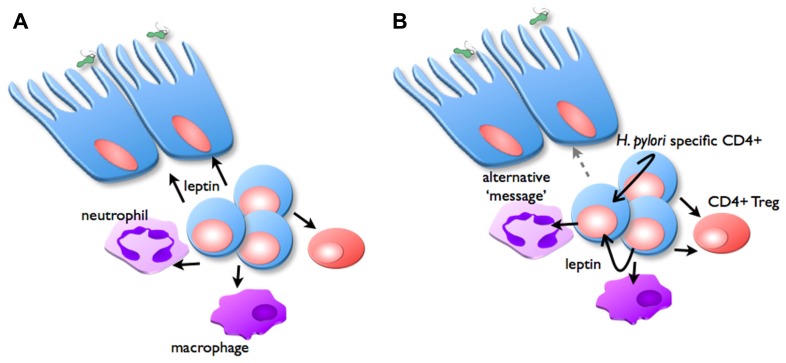
**Possible role of leptin in inflammation and vaccine-induced protection.** In the vaccinated stomach, *H. pylori* challenge triggers CD4^+^ T cells, neutrophils, and macrophages to infiltrate the mucosa, proportions of T_reg_ are reduced. Two possible mechanisms are proposed for the manner in which leptin signaling is important for regulating this inflammation: **(A)** CD4^+^ T cells secrete leptin (black arrows), which acts as the message to stimulate epithelial cells to facilitate protective responses. **(B)** Alternatively, CD4^+^ T cells secrete leptin, which acts in an autocrine manner to stimulate their own proliferation, and suppress T_reg_ function. Leptin also affects macrophages and neutrophil function, depending on expression of specific LepR isoforms. This signaling induces secretion of other messages (gray arrow), which in turn stimulate epithelial cells to facilitate protective responses.

It remains to be demonstrated whether leptin receptor expression at the level of lymphocytes (CD4^+^ T cells or T_reg_), or indeed the gastric epithelium are most important in mediating the protective effects of vaccination. It is conceivable that leptin secreted by T cells may signal via LepR on gastric epithelial cells, and therefore mediate a protective response (**Figure 4**). Alternatively, LepR signaling on CD4^+^ T cells and/or T_reg_ may regulate inflammation and therefore the protective response. We are currently investigating these possibilities (Becher et al., in preparation). If these interactions are shown to play a significant role, it will be interesting to determine the extent to which they interact with the previously demonstrated role for IL-17 in protection and inflammation in *H. pylori* infection (Velin et al., 2009; Flach et al., 2011).

Our current studies are addressing a series of questions which we believe will provide a “road map” for testing the hypothesis:

- Are the effects of leptin signaling mediated by direct action on epithelial cells, or immune (bone marrow-derived) cells the gastric mucosa?
- Does *local* leptin production can affect T cell and T_reg_ function in the mucosa? Are the effects on these cells direct or indirect? Or is systemically produced leptin required?
- And finally, and more speculatively is there a relationship between the effects of IL-17 and leptin in the stomach? It is possible that these mediators are part of the same local “program,” or that their effects are specific and sequential.

In conclusion, we have provided evidence for the proposal that leptin has an important role in regulating T_reg_ and CD4^+^ T cell function in the gastric tissue. More detailed studies testing these hypotheses are underway and will ultimately clarify this. In the interim, we believe at least that this adipokine should be considered as an important player in the control of gastric inflammation.

## Conflict of Interest Statement

The authors declare that the research was conducted in the absence of any commercial or financial relationships that could be construed as a potential conflict of interest.

## USE OF EXPERIMENTAL ANIMALS

All studies were performed with the approval of the University of Melbourne Animal Ethical and Experimentation Committee, in accordance with NHMRC guidelines.
